# Clinical characteristics and variant analyses of transient infantile hypertriglyceridemia related to GPD1 gene

**DOI:** 10.3389/fgene.2022.916672

**Published:** 2022-08-16

**Authors:** Jun Wang, Xinrong Sun, Lianying Jiao, Zhengtao Xiao, Farooq Riaz, Yufeng Zhang, Pengfei Xu, Ruiqing Liu, Tiantian Tang, Meiqi Liu, Dongmin Li

**Affiliations:** ^1^ Department of Biochemistry and Molecular Biology, School of Basic Medical Science, Xi’an Jiaotong University Health Science Center, Xi’an, China; ^2^ Second Department of Infectious Disease, Children’s Hospital Affiliated to Xi’an Jiaotong University, Xi’an, China; ^3^ Center for Cancer Immunology Research, Shenzhen Institutes of Advanced Technology, Chinese Academy of Sciences, Shenzhen, China

**Keywords:** glycerol-3-phosphate dehydrogenase 1 (GPD1), transient infantile hypertriglyceridemia (HTGTI), hypertriglyceridemia, hepatic steatosis, hepatomegaly

## Abstract

**Objective** **:** Our study aims to summarize and analyze the clinical characteristics of transient infantile hypertriglyceridemia (HTGTI) and variants in the glycerol-3-phosphate dehydrogenase 1 (*GPD1*) gene and the effect of HTGTI on the protein structure of *GPD1*.

**Methods:** Retrospective analysis, using the general data, symptoms, signs, and auxiliary examinations, was performed on patients with HTGTI, which were confirmed by genetic testing in our hospital and reported cases online. The clinical data were analyzed using statistical and bioinformatic approaches.

**Results:** A total of 31 genetically confirmed HTGTI patients were collected from our hospital and cases reported in the literature. The clinical manifestations showed the median age of onset was 6.0 (1.9, 12.0) months. All the patients had normal psychiatric status, but 22.6% of them presented growth retardation and short stature, 93.5% had hepatomegaly, and 16.1% had splenomegaly. Just a few children were reported with jaundice, cholestasis, and obesity (3.2–6.5%). The laboratory investigations showed that 96.8% of them had hypertriglyceridemia (HTG) with a median level of 3.1 (2.1, 5.5) mmol/L, but only 30.0% had returned to normal during follow-up. In addition, 93.5% of patients had elevated alanine aminotransferase (ALT) with an average level of 92.1 ± 43.5 U/L, while 38.7% had hypercholesterolemia. Upon abdominal imaging, all patients presented fatty liver and liver steatosis, with 66.7% of patients showing hepatic fibrosis. Statistical differences in triglyceride (TG) level were observed in the ≤6 months group compared with the older groups and in the 13 months to 6 years group with >6 years group (*H* = 22.02, *P* < 0.05). The restricted cubic spline model showed that severe HTG decreased in the early stage of infants to the normal level; however, it rebounded again to a mild or moderate level after the following days. The genetic test revealed that the main variant types of the *GPD1* gene were missense variants (51.6%), followed by splicing variants (35.5%) and nonsense variants (12.9%). Of patients, 87.1% had homozygous variants, with the most frequent loci being c.361-1G > C and c.895G > A.

**Conclusion:** The common manifestations of HTGTI were HTG, hepatomegaly, elevated liver transaminases, and hepatic steatosis in early infancy. However, the recurrence of aberrant HTG may pose long-term detrimental effects on HTGTI patients.

## Introduction

Hypertriglyceridemia (HTG), which refers to elevated fasting triglyceride (TG) that exceeds 1.7 mmol/L, is a relatively prevalent condition in clinical practice (Berglund et al., 2012). It affects 10–20% of the adult population with considerable interregional variation in the incidence rate (Parhofer and Laufs 2019; Laufs et al., 2020). In addition, HTG is a significant risk factor for obesity, atherosclerotic vascular diseases, type 2 diabetes, acute pancreatitis, and other related metabolic syndromes. Based on the genetic and nongenetic etiologies of HTG, the current categorization of HTG segregates primary and secondary causes for each category (Brunzell 2007; Hegele et al., 2014). HTG is considered a complicated phenotype resulting from the complex regulatory networks of multiple susceptibility genes (common and rare genetic variants) and environmental factors (Johansen et al., 2010; Hegele et al., 2014). The single-gene variants of primary HTG identified by gene sequencing include *LPL*, *APOC2*, *APOA5*, *LMF1*, *GPIHBP1*, and *GPD1*. Severe HTG (TG > 10.0 mmol/L) is more likely to be associated with single-gene variation, especially in infants and adolescents (Laufs et al., 2020; Shah and Wilson, 2020).

Transient infantile hypertriglyceridemia (HTGTI) is a sporadic autosomal recessive hereditary disease with lipid metabolic disorder. It is caused by the inactivation and variant of glycerol-3-phosphate dehydrogenase 1 (GPD1, MIM138420) located on chromosome 12q12-q13, which is also a crucial molecular cause of congenital HTG in infants. The *GPD1* gene encodes intracytoplasmic NAD-dependent GPD1, which plays an essential role in lipid and carbohydrate metabolism by catalyzing the reversible redox reaction of dihydroxyacetone phosphate (DHAP) and reduced nicotine adenine dinucleotide (NADH) to glycerol-3-phosphate (G3P) and NAD^+^ (Ou et al., 2006). To date, 31 genetically confirmed patients have been reported worldwide in 10 case reports. The most common clinical manifestations were transient hypertriglyceridemia, hepatomegaly, elevated liver transaminases, and hepatic steatosis in early infancy (Basel-Vanagaite et al., 2012; Joshi et al., 2014; Dionisi-Vici et al., 2016; Li et al., 2017; Li et al., 2018; Matarazzo et al., 2020; Xie et al., 2020; Kumar and Sharma 2021; Tesarova et al., 2021; Wang et al., 2021). In some patients, HTG is normalized with age. However, during follow-up, other patients may exhibit mild HTG accompanied by abnormal liver transaminases, fatty liver, and even cirrhotic manifestations. However, there is inadequate understanding of the multiple clinical phenotypes and long-term prognosis of HTG, and it requires detailed systematic and comprehensive studies. This article aims to summarize and analyze the clinical characteristics of HTGTI and variants in the *GPD1* gene, which will serve as a reference for further clinical diagnosis, therapy, and research.

## Materials and methods

### Source of cases and search strategy

One confirmed case of HTGTI was from our hospital. Meanwhile, to retrieve the other subjects from previously published reports, we searched the relevant literature about HTGTI caused by the *GPD1* gene variant in databases. The search was conducted up to September 2021 using the following electronic databases: Medline, Cochrane Library, EMBASE, PubMed, and Web of Science. The search terms used to retrieve data were “Glycerol-3-Phosphate Dehydrogenase (NAD^+^),” “Glycerol-3-phosphate dehydrogenase 1,” “*GPD1*,” “HTGTI,” or “transient infantile hypertriglyceridemia,” in association with a list of sensitive terms to search for experimental studies or case reports about humans. In addition, the reference lists provided in the selected articles were hand-searched to investigate further relevant studies.

### Data extraction

The general characteristics of studies were extracted, including the age, gender, nationality, age of onset, clinical manifestations, laboratory and genetic examination results, treatments, and follow-up of patients. The collected data were collated and summarized in tables and figures.

### Sequencing study and analysis

The genetic testing was performed after obtaining written informed consent from our patient’s parents, and the peripheral venous blood of the proband and her parents were collected for genetic testing using next generation of whole genomic DNA(Wang et al., 2021). The captured libraries were sequenced using Illumina HiSeq, analyzed using clinic sequence analyzer from WuXiNextCODE (China), and tested using the SureSelect Human All Exon V5 kit (Agilent Technologies, Inc.), Illumina Cluster kit, and SBS kit according to the manufacturer’s standard procedure. The average sequencing depth of exome target sequencing is greater or equal than 90×, in which 95% of the target sequencing is over 20×. Base calling was carried out for all sequences.

In the second round, sequencing data were mainly analyzed using Sentieon. The reads were mapped to the reference of Sentieon BWA and UCSC hg19. The sequencing depth and variants prediction of each base were extracted from all genomic sequencing data. Variants were noted by VEP (Variant Effect Predictor, Ensembl73). Three databases including ClinVar, OMM, and HGMD were employed to filter known and possible pathogenic variants, and other tools were adopted to predict the function of missense mutation and to note non-coding sequence. Large-scale sequencing databases including 1000 Genome Project, Exome Sequencing Project, Exome Aggregation Consortium, and Genome Aggregation Database of Tokyo and the Netherlands, were used to filter frequent variants. Every variant was assessed with clinic sequence analyzer, named in accordance with the guidelines of Human Genome Variation Society, and classified based on the standards and guidelines for the interpretation of sequence variants by American College of Medical Genetics and Genomics. Variants were verified in the patient and her parents by Sanger sequencing. The other children with HTGTI also underwent similar tests with gene sequencing, which were described in the above case reports.

### Structural diagram of glycerol-3-phosphate dehydrogenase 1 gene and protein

The nucleotide reference sequence of the *GPD1* gene was derived from NCBI (transcript: NM_005276.4). The crystal structure of human GPD1 was retrieved from the Protein Data Bank; its primary citation of related structures is 1X0V. The picture of the gene structure was prepared using illustrator for biological sequences (IBS) (Liu et al., 2015), and the protein structures of GPD1 were drawn using PyMOL software (The PyMOL Molecular Graphics System, http://www.pymol.org).

### Statistical analyses

Excel 2019 software and SPSS 18.0 statistical software were used for statistical analysis. The normally distributed data were expressed as mean ± SD, while the nonnormally distributed variables were expressed as median M (Q1, Q3); however, the count data were expressed as percentages (%). Normal distributed continuous variables were analyzed using either one-way analysis of variance or the LSD test. Differences in the characteristics between groups were analyzed using the Kruskal–Wallis test with the Dunn *post hoc* tests (for continuous variables, R package FSA) or the χ^2^ tests with *post hoc* tests (for categorical variables, R package fifer). We also used restricted cubic spline models fitted for the hazards models of TG with age using R with ggplot2 (Xu et al., 2021). Values of *p* less than 0.05 were considered statistically significant.

## Results

### General information of patients

**FIGURE 1:**
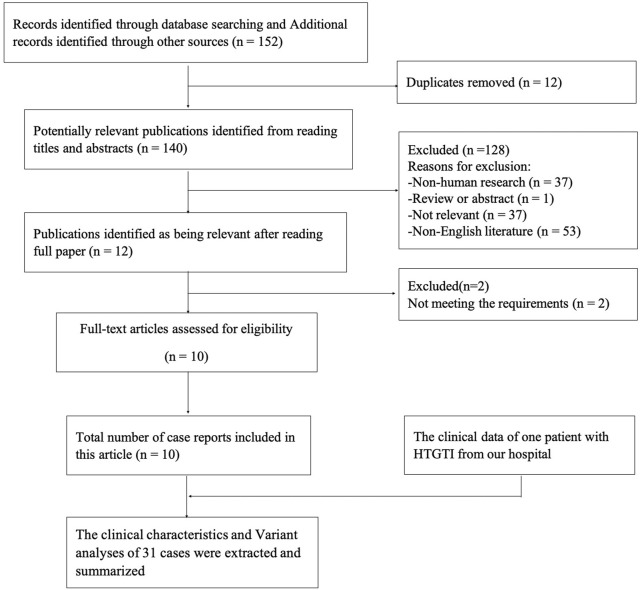
The flow diagram of the research process.

Our search strategy identified 152 potentially relevant articles from the online databases. After removing duplicates, 140 studies remained. Reading the titles and abstracts of these references led us to exclude a further 128 identified articles that did not meet the inclusion criteria. After reading the full text of the remaining 12 articles, 2 were excluded because of the lack of relevant information. In the end, a total of 10 case reports were retrieved related to HTGTI with GPD1 variants from the above-mentioned medical research databases, including 31 genetically confirmed patients. The selection process is shown in Figure 1. Among these patients, 13 (61.9%) and 8 (38.1%) were male and female, respectively, with a male-to-female ratio of 1.6:1; however, 10 cases lack the related information. Meanwhile, their onset age was widely variable, ranging from 0.0 to 7.0 years, with a median age of 6.0 (1.9, 12.0) months. Ethnic distribution shows that 12 patients were Arabs; 9 patients were Roma; 4 patients were Chinese; 2 patients were Italian; 1 patient each from Russia, Caucasus, and India; and only 1 patient was unknown. In addition, 12 patients come from families with a high degree of consanguinity, 5 patients were from nonconsanguineous families, and 14 patients were unknown. Just one patient had a family history of fatty liver in parents, one had a morbidly obese father, and others were unknown.

### Clinical manifestation

**FIGURE 2:**
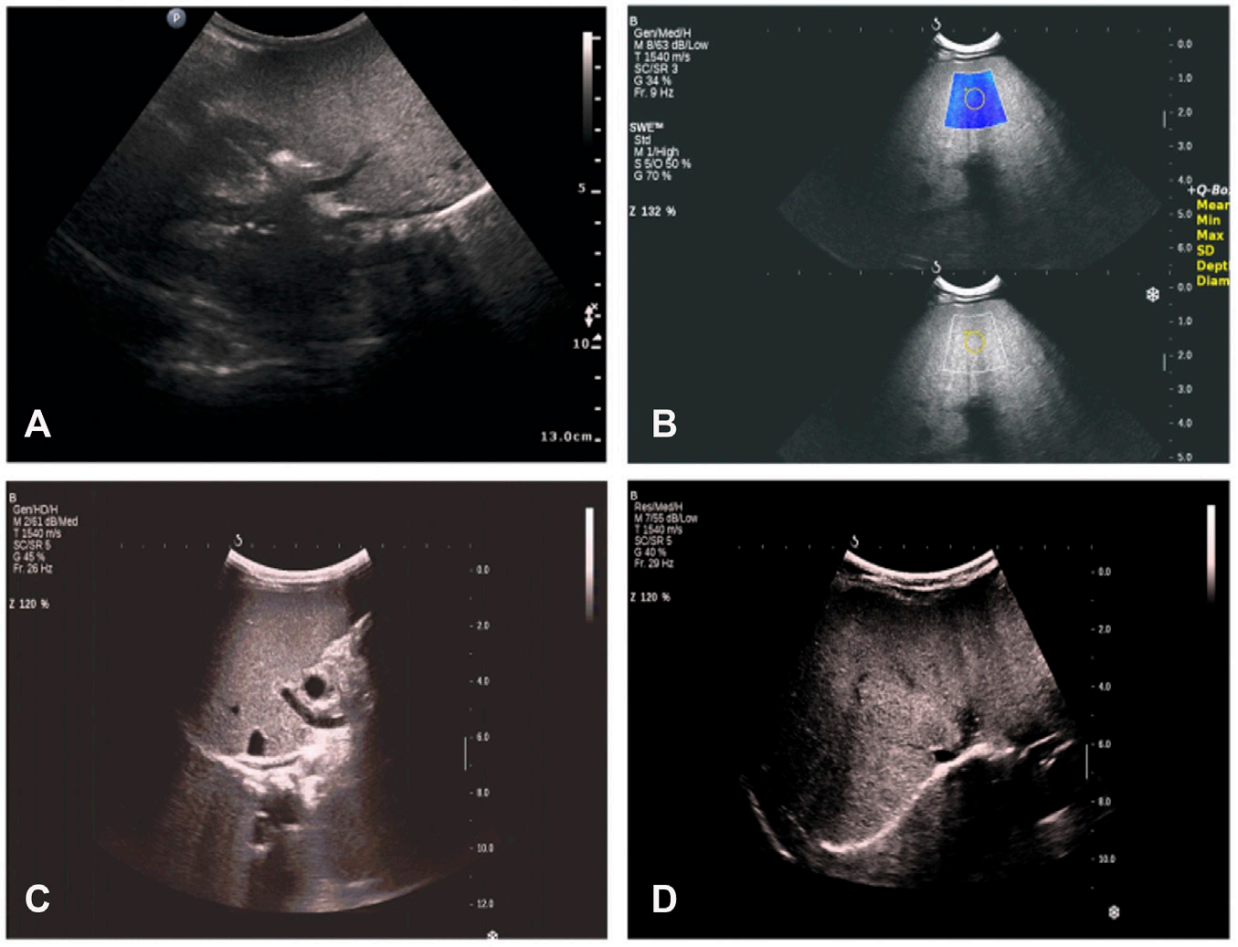
Radio findings in proband with glycerol-3-phosphate dehydrogenase 1 (GPD1) mutation from our hospital. **(A)** at the age of 1 month 26 days: hepatosplenomegaly, thicker parenchyma echo spots, slightly coarse gallbladder wall, and gallbladder contraction rate was less than 50% after 1 h of breastfeeding. **(B)** at the age of 2 months 4 days: hepatosplenomegaly, thinner parenchyma echo spots, strengthened echo, thicker and coarse gallbladder wall, and liver tissue elastic modulus was approximately 15 kPa. **(C)** at the age of 7 months 10 days: hepatosplenomegaly, stronger and diffused echo, and liver tissue elastic modulus was approximately 17 kPa. **(D)** at the age of 1 year and 4 months: fatty liver change; no obvious abnormality of gallbladder, pancreas, liver, and spleen; liver tissue elastic modulus was approximately 14.3 kPa.

We analyzed the clinical characteristics of these 31 patients with HTGTI. According to the data from the beginning of the visit to the last follow-up, the median follow-up time was 4.1 (0.9, 12.6) years. The age of the original diagnosis of HTGTI was 0.0–24.0 months, and the median age was 6.0 (1.8–10.0) months. All the patients had normal psychiatric status, but 22.6% of them presented with growth retardation and short stature. HTG was identified in 96.8% (30/31) of patients, with elevation ranging from 1.9 to 70.6 mmol/L, and the median level was 3.1 (2.1, 5.5) mmol/L. However, the HTG level of only 30.0% (6/20) of children returned to normal during follow-up. Hepatomegaly, which was palpated less than 10 cm below the costal margin, was observed in 93.5% (29/31) of patients. Splenomegaly, which was palpated less than 5 cm below the costal margin, was reported in 16.1% (5/31) of patients. A few patients presented with jaundice (6.5%), elevated cholestasis (3.2%), hypoglycemia (9.7%), and obesity (3.2%). Imaging analysis showed that 100.0% of patients had fatty liver (Figure 2). The liver biopsy specimens from 15 patients presented steatosis, of which 66.7% (10/15) were accompanied by hepatic fibrosis. Blood screenings were normal, but 6.7% (2/30) of urine screenings reported urine dicarboxylic acids. Of patients, 87.1% had homozygous variants, with the most frequent loci being c.361-1G > C and c.895G > A (Table 1).

**TABLE 1:** GPD1 gene variant and phenotype of 31 patients with HTGTI.

References	Descent	Number of patients	c.DNA variant		Hom/Het	Amino acid change	Variant region	Variant type	Main phenotype of pathogenic variant
			Maternal	Paternal					
Basel-Vanagaite et al. (2012)	Israeli-Arab	10	c.361-1G > C	c.361-1G > C	Homozygous	Ile119fsX94	Intro 3	Splicing variant	Hepatomegaly, fatty liver, HTG, 4/10 had short stature, vomit, slow weight gain, elevated ALT + GGT
Joshi et al. (2014)	Caucasian	1	c.686G > A	a small deletion	Compound heterozygous	R229Q	Exon 6	Missense variant	Hepatomegaly, fatty liver, HTG, maldevelopment, vomit, elevated transaminase + GGT + TC ^[11]^
Dionisi-Vici et al. (2016)	Arab-Muslim	1	c.806G > A	c.806G > A	Homozygous	Arg269Gln	Exon 6	Missense variant	Hepatomegaly, fatty liver, HTG, elevated transaminase, recurrent fasting hypoglycemia
	NA	1	c.361-1G > C	c.361-1G > C	Homozygous	Ile119fsX94	Intro 3	Splicing variant	Hepatomegaly, fatty liver, HTG, elevated transaminase + GGT + ALP
	Italian	1	c.640T > C	c.640T > C	Homozygous	Cys214Arg	Exon 6	Missense variant	Hepatosplenomegaly, fatty liver, HTG, elevated transaminase + total bile acid (TBA), urine dicarboxylic acids, minimal lesion nephropathy
	Italian	1	c.640T > C	c.640T > C	Homozygous	Cys214Arg	Exon 6	Missense variant	Hepatomegaly, fatty liver, HTG, elevated transaminase + TC + TBA
Li et al. (2017)	Chinese	1	c.820G > A	c.220–2A > G	Compound heterozygous	Ala274Thr	Exon 6 and 3	Missense variant	Obesity, fatty liver, short stature, insulin resistance, dermal abnormalities, elevated dehydroepiandrosterone sulfate and lipoprotein-α(LP-α), normal lipid
Li et al. (2018)	Chinese	1	c.523C > T	c.523C > T	Homozygous	Q175*	Exon 5	Nonsense variant	Hepatomegaly, fatty liver, HTG, elevated transaminase + GGT + TBA
Matarazzo et al. (2020)	Russian	1	c.895G > A	c.895G > A	Homozygous	G299R	Exon 7	Missense variant	Hepatomegaly, fatty liver, cirrhosis, HTG, elevated transaminase + TBA
Wang et al. (2021)	Chinese	1	c.901G > T	a short deficiency	Compound heterozygous	E301X	Exon 7	Nonsense variant	Hepatomegaly, fatty liver, jaundice, cirrhosis, elevated transaminase + GGT + TBA, urine dicarboxylic acids
Xie et al. (2021)	Chinese	1	c.901G > T	c.931C > T	Heterozygous	E301X + Q311X	Exon 7	Nonsense variant	Hepatomegaly, HTG, elevated transaminase
Kumar and Sharma (2021)	Indian	1	c.500G > A	c.500G > A	Homozygous	Gly167Asp	Exon 5	Missense variant	abdominal distention, Hepatomegaly, HTG, slight Splenomegaly, elevated ALP
Tesarova et al. (2021)	Romani	9	c.895G > A	c.895G > A	Homozygous	Gly299Arg	Exon 7	Missense variant	9 of 10 had hepatomegaly, elevated transaminase, low-normal growth; 2 had mild repeated hypoglycemia
	Palestinian Arab	1	c.116G > A	c.116G > A	Homozygous	Trp39*	Exon 2	Nonsense variant	

Items: HTG, hypertriglyceridemia; ALT, alanine aminotransferase; GGT, gamma-glutamyl transpeptidase; TC, total cholesterol; ALP, alkaline phosphatase; TBA, total bile acid.

**FIGURE 3:**
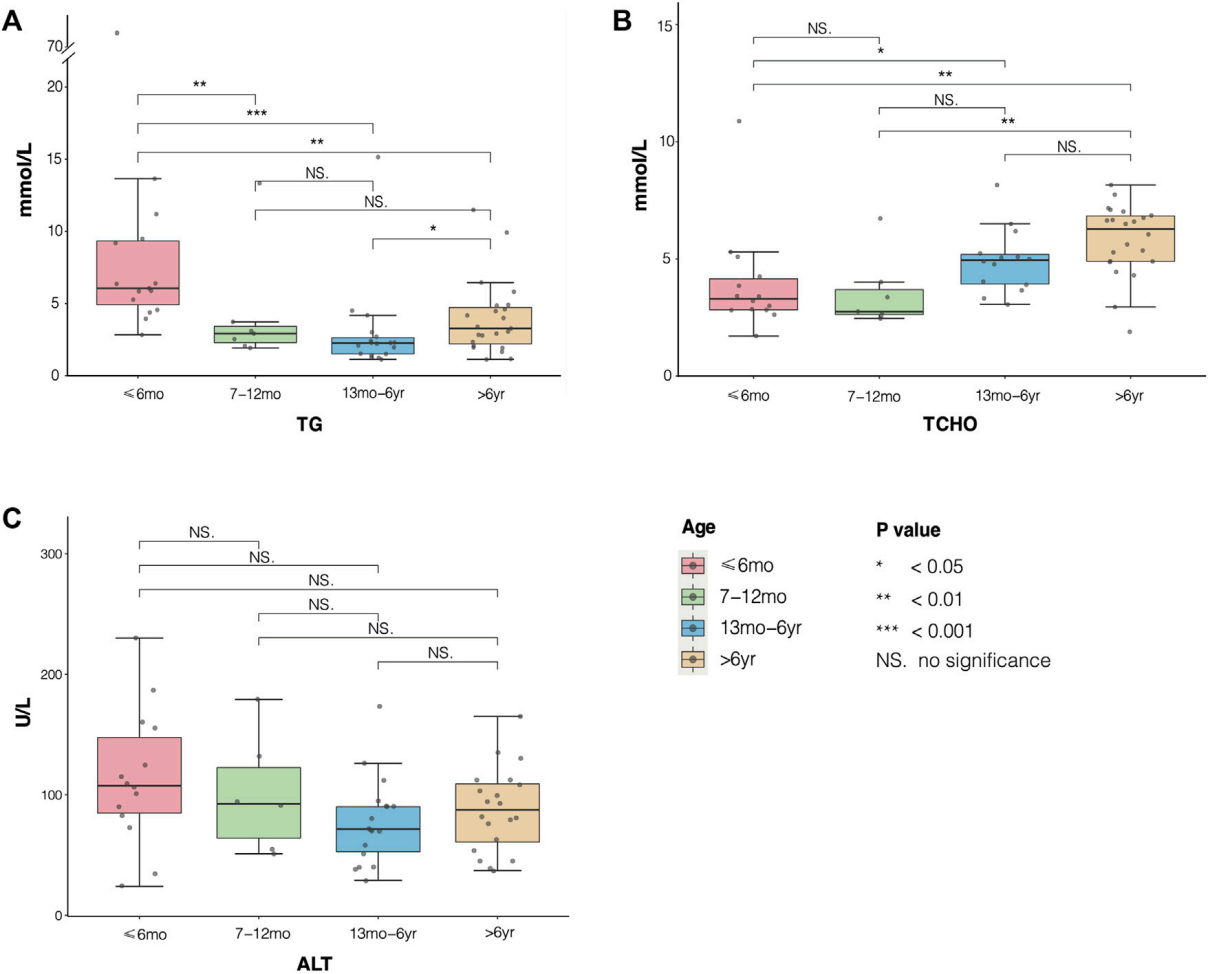
Distribution of serum triglyceride (TG), TCHO, and alanine aminotransferase (ALT) in different age groups of patients with transient infantile hypertriglyceridemia (HTGTI). The boxplots showed median and interquartile level values of TC, TCHO, and ALT in the four age groups. **(A)** statistical differences in TG were shown in group ≤ 6months compared with the other three groups, and group 13 months to 6 years with group >6 years (*P* < 0.05). **(B)** significant differences in TCHO were observed in group ≤6 months compared with group 13 months to 6 years and group >6 years, and group 7–12 months with group 6 years (all P < 0.05). **(C)** there were no significant differences in serum ALT among the four groups (all P > 0.05).

Furthermore, to analyze the average levels of serum TG, total cholesterol (TC), and alanine aminotransferase (ALT) of 31 patients, we divided them into four groups: younger than 6 months (≤6 months group), 7–12 months (7–12 months group), 13 months to 6 years (13 months to 6 years group), and older than 6 years (>6 years group) (Table 2; Figure 3). The median levels of TG, TCHO, and ALT were 5.85 (4.16, 9.33) mmol/L, 4.90 (3.13, 6.50) mmol/L, and 90.00 (57.25, 112.00) U/L, respectively. Next, we drew boxplots based on the laboratory data from the reported cases. As shown in Figure 3, the median level of TG was highest in the ≤6 months group. In contrast, the level of TG tended to decrease with age but rebounded again in the >6 years group. Furthermore, statistical differences (using the Kruskal–Wallis test with Dunn’s test) in TG levels were observed among four groups. Results indicated that the ≤6 months group showed a significant increase in TG levels compared to the other three groups, and the 13 months to 6 years group was comparable with the >6 years group (*H* = 22.02, *P* < 0.05). The level of TCHO was lower in the 7–12 month age group than in the ≤6 month age group, but it was higher in the 13 months to 6 years group and the >6 years group. Significant differences were also observed in the ≤6 months group compared with that of the >6 years group, and the 7–12 months group showed significant difference compared with the >6 years group (*H* = 17.77, all *P* < 0.05). No significant differences were observed in the levels of serum ALT between or within the four age groups (*H* = 5.35, all *P* > 0.05).

**TABLE 2:** Differential analysis of TC, TCHO, and ALT in different age groups. Significant differences existed in TG and TCHO in different age groups (*P* < 0.05), but there were no statistical differences in ALT between four groups (*P* > 0.05) (a, b, c, and d are used to represent the statistical difference between groups with the same superscript letter).

Items	subgroup	Median/mean	H-value	*p-*value
TG	≤6 months	5.85 (4.16, 9.33)^a^	22.023	0.000
	7–12 months	2.92 (2.05, 3.73)^a^		
	13 months to 6 years	2.28 (1.64, 2.93) ^ab^		
	>6 years	3.27 (2.07, 4.86) ^ab^		
TCHO	≤6 months	3.30 (2.82, 4.46)^c^	17.767	0.001
	7–12 months	2.75 (2.56, 4.01)^d^		
	13 months to 6 years	4.95 (3.84, 5.47)		
	>6 years	6.28 (4.89, 6.90) ^cd^		
ALT	≤6 months	107.50 (80.5, 156.25)	5.35	0.148
	7–12 months	92.50 (54.00, 143.75)		
	13 months to 6 years	71.50 (48.25, 91.25)		
	>6 years	87.5 (54.00, 108.00)		

### Correlation between blood triglyceride and age using restricted cubic spline

**FIGURE 4:**
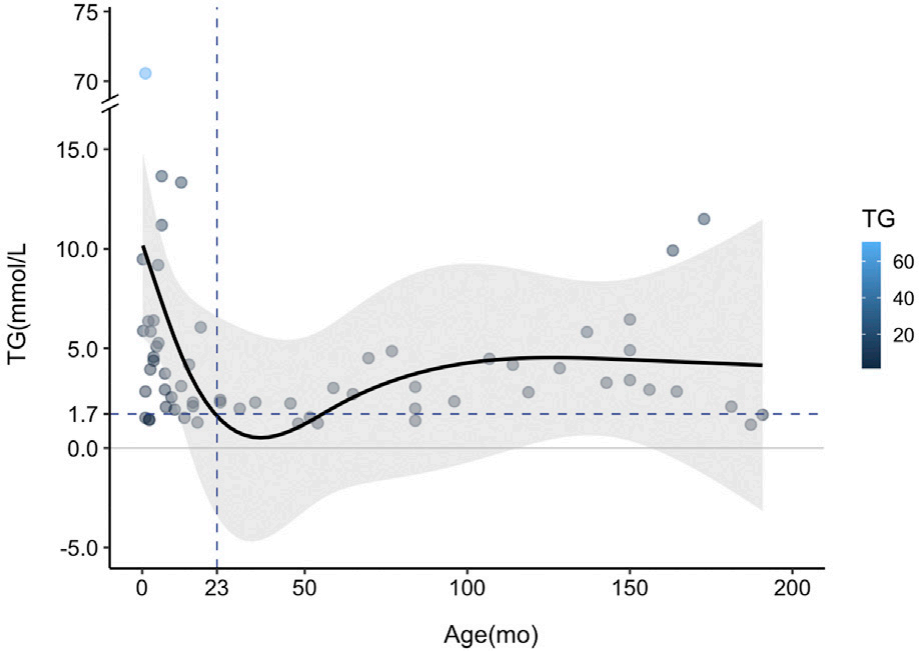
Curve fitting of serum triglyceride (TG) changing trend with age in patients with transient infantile hypertriglyceridemia (HTGTI). Restricted cubic spline (RCS) with four knots was used to model the association of serum TG with age. The curve shows a good simulation of the correlation between this binary (*p* < 0.001). The TG level decreased sharply from the peak point in the early age of infants to the normal value (1.7 mmol/L) but rebounded during follow-up, especially after approximately 50 months old.

To further analyze the TG trends with the age of HTGTI patients, we used restricted cubic spline (RCS) with four knots at 3, 12, 60, and 150 months to flexibly model the association of serum TG with age (Figure 4). The association between the levels of TG on a continuous scale and the age of these patients was U-shaped. This curve shows a good simulation of the correlation between these binary variables (P for nonlinearity <0.001). It predicted that the high level of TG in the early stage of infants decreased sharply with age and became normal (1.7 mmol/L) at the age of 23 months. Still, TG level rebounded again after infants reached 50 months old and maintained at relatively stable levels. Considering that all patients with HTGTI exhibited HTG, steatosis, and even hepatic fibrosis during the onset and follow-up period, HTG does not mean a transient phenomenon only in infancy. Meanwhile, we also believe that rebounded or sustained elevation of TG was associated with an increased risk of disease. Although the severe HTG may be transient in infancy in HTGTI, mild to moderate dyslipidemia is likely to be persistent in many of these patients. Even though there is still a lack of abundant and longer-term follow-up data, this situation may be associated with future adverse health outcomes.

### Analysis of glycerol-3-phosphate dehydrogenase 1 genetic test

**FIGURE 5:**
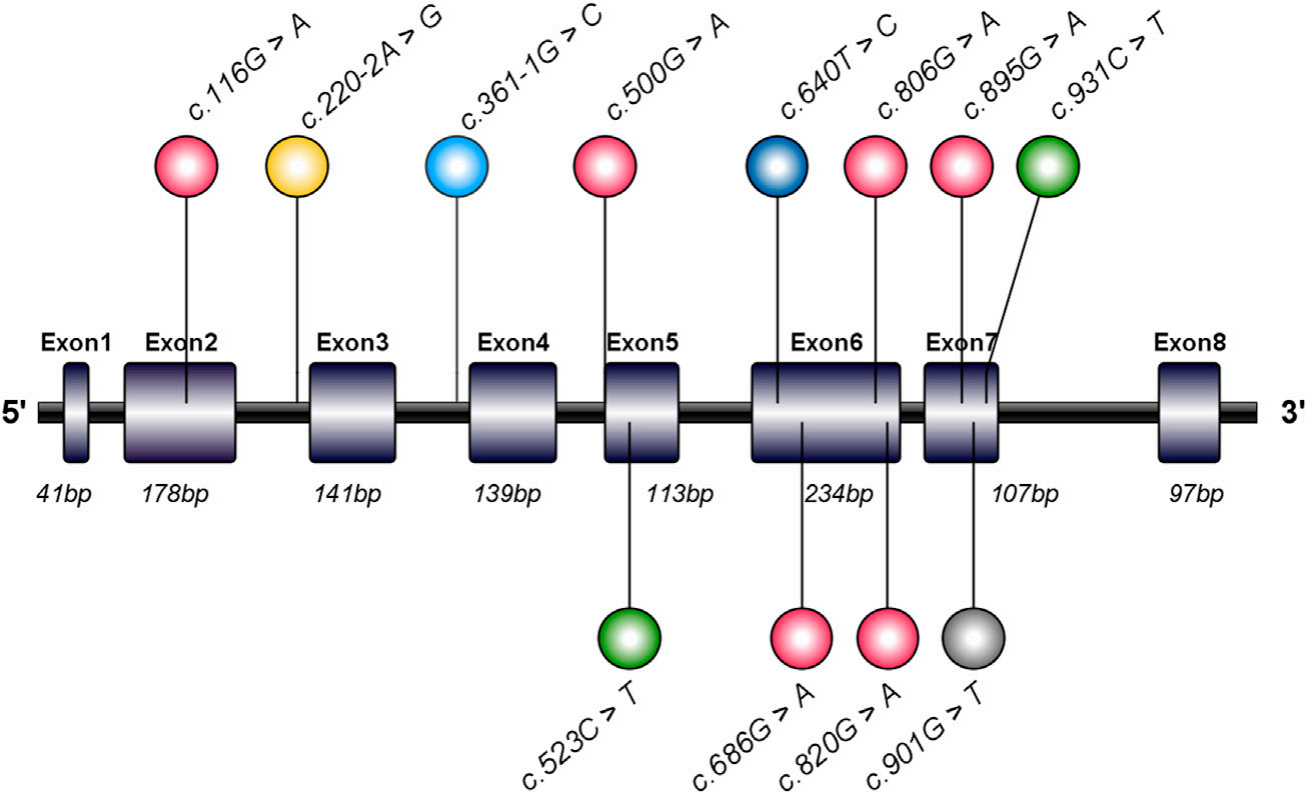
Schematic diagram and mutation sites of glycerol-3-phosphate dehydrogenase 1 (*GPD1*) gene in transient infantile hypertriglyceridemia (HTGTI) patients. The human GPD1 contains eight exons and encodes a protein consisting of 349 amino acid residues. This image illustrates the spectrum of GPD1 mutation sites associated with HTGTI from the reported cases, marked in the lollipop style, including 10 mutation sites on exons and 2 on introns. This spectrum was generated using illustrator for biological sequences (IBS).

The *GPD1* gene (NG_032168.1, NC_000012.12, including 7306 bases, gene ID: 2819) found in the GenBank gene database shows that the *GPD1* gene has a total length of 7,306 bp and is located on the long arm of human chromosome 12 (12q13.12). *GPD1* includes eight exons and seven introns, while coding sequence (CDS) encodes 349 amino acid residues (Figure 5). Analysis of *GPD1* gene variant loci and clinical phenotype (Table 1) shows that the variant sites of the *GPD1* gene were mainly missense variants (16/31), followed by splicing variants (11/31) and nonsense variants (4/31). These reported variants resulted in the dysfunction of GPD1.

**FIGURE 6:**
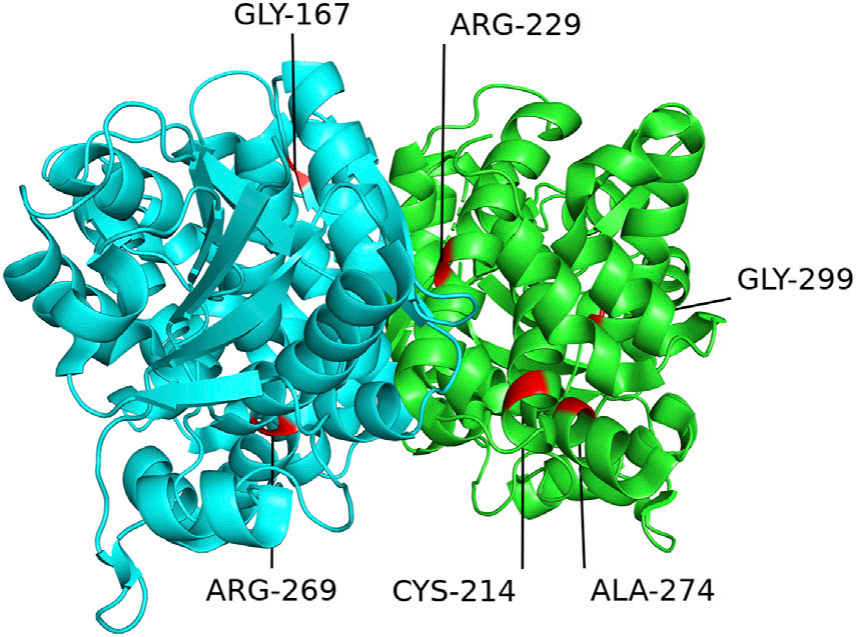
Schematic diagram of protein structure of mutation loci of the glycerol-3-phosphate dehydrogenase 1 (*GPD1*) gene. This diagram shows the location of the GPD1 missense mutation sites in the protein structure (dimer) in patients with transient infantile hypertriglyceridemia (HTGTI): it mainly exists in the dimer binding surface (R229Q), active site (R269Q), helical bundle (C214R, A274T), near active site (Q299R), and β-folding (G167D). These protein structures of GPD1 were drawn using PyMOL software.

**FIGURE 7:**
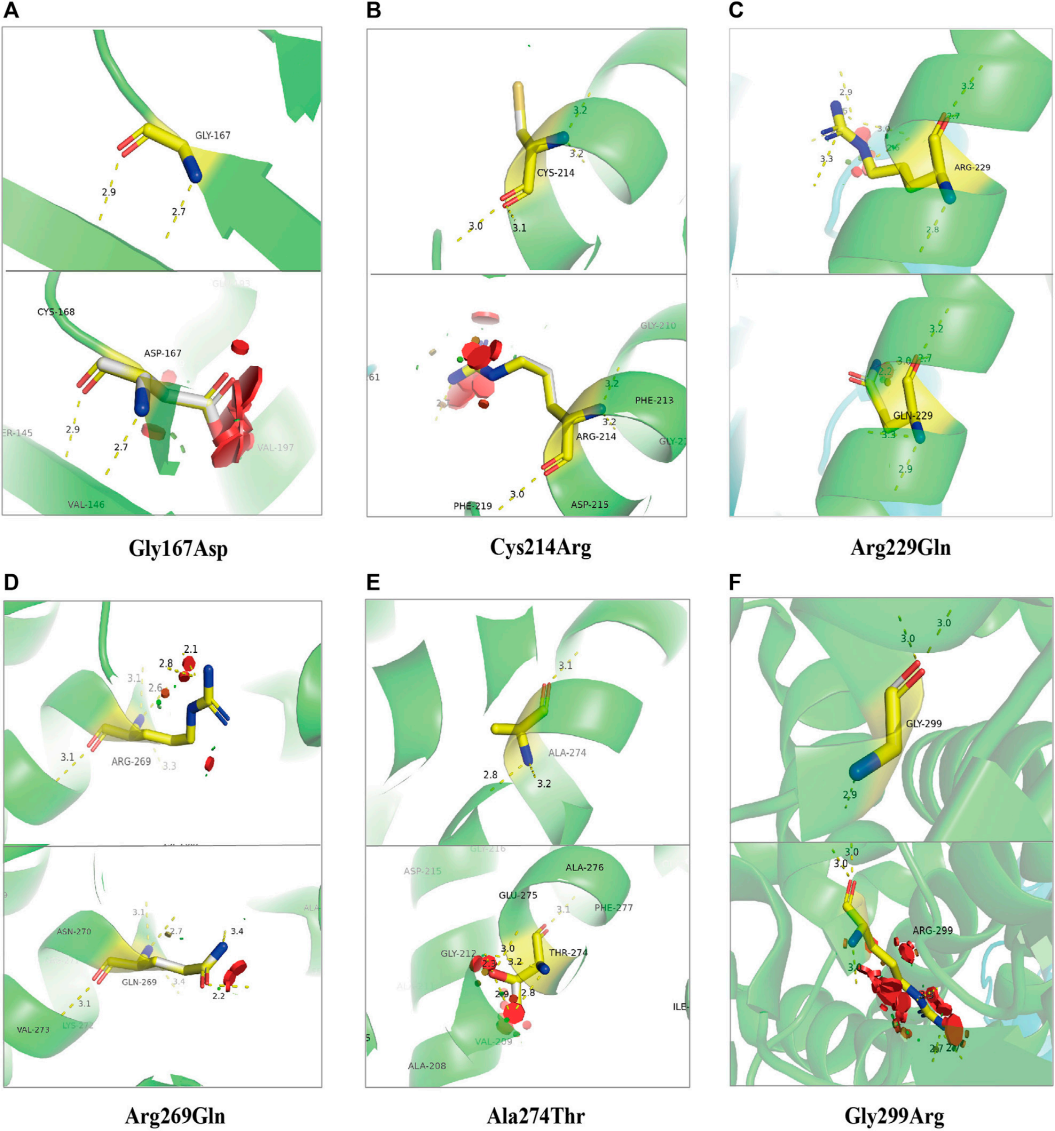
Stimulation diagram of amino acid/protein three-dimensional structure changes caused by missense variants of *GPD1* gene. They exist in the β-folding [**(A)**. Gly167Asp], helical bundle [**(B)**. Cys214Arg and **(E)**. Ala274Thr], the dimer binding surface [**(C)**. Arg229Gln], active site [**(D)**. Arg269Gln and **(F)**. Gly299Arg]. We can see these tertiary structural changes in the spatial strcture of amino acid disability before and after missense variants at these sites. This picture was drawn using PyMOL software.

Figure 6 and Figure 7 show the schematic diagrams of the protein structure (dimer) of GPD1 missense variant loci, which mainly exists in the dimer binding surface (R229Q), active site (R269Q), helical bundle (C214R, A274T), near active site (Q299R), and β-folding (G167D), in patients with HTGTI. Arg269, as a conserved active site of the ternary complex of GPD1/DHAP/NAD^+^, is in direct contact with the substrate and provides 3 of 11 hydrogen bonds in the dimer interface. Gly299 constitutes the other fixed point of the three-dimensional structure of the binary complex of GPD1 with NAD^+^ (Ou et al., 2006). Furthermore, we can see the simulation diagram of amino acid/protein three-dimensional structural changes caused by a missense variant of the *GPD1* gene. These six missense variants of the *GPD1* gene result in the substitution of one amino acid for another. We can see these tertiary structural changes in the spatial structure of amino acid disability before and after missense variants at these sites. The defective protein assembly and connective enzyme structure may lead to loss of GPD1 enzyme activity.

At present, 10 types of GPD1 variants in HTGTI patients have been reported. As this disease is a very rare genetic metabolic disease in children, the specific carrying frequencies of many mutation sites of the *GPD1* gene in the population was not available in the public databases such as Genecards and dbSNP. The most frequent variant types of GPD1 are c.361-1G > C (35.5%) and c.895G > A (32.3%). The odds ratio (OR) of c.361-1G > C to c.895G > A (32.3%) is 1.15 (95% confidence interval (CI) 0.40–3.31, *P* = 0.39), and the OR of c.361-1G > C to c.640T > C and c.901G > T was 7.98 (95% CI 1.59–39.9, *P* = 0.006); moreover, the OR of c.361-1G > C to c.686G > A, c.806G > A, c.820G > A, c.220–2A > G, c.523C > T, c.931C > T, c.500G > A, and c.116G > A was 16.5 (95% CI 1.97–138.0, *P* = 0.005).

## Discussion

Transient infantile hypertriglyceridemia (HTGTI) was first reported by Basel-Vanagaite et al. (2012). They explicitly showed primary dyslipidemia, a lipid metabolism disorder, in early infancy is caused by a monogenic variant in the *GPD1* gene. Among the reported 31 patients all over the world, the typical clinical manifestations are hypertriglyceridemia, hepatomegaly, elevated transaminase, hepatic steatosis, and early-stage hepatic fibrosis. Additional heterogeneous phenotypes include prolonged jaundice, cholestasis, fasting ketosis, hypoglycemia, insulin resistance, obesity, kidney disease, and growth retardation. The cases of HTGTI have been reported in different regions of the world and among various ethnic groups. It suggests that there may be no regional and ethnic differences in prevalence. Although the ratio of males to females is 1.6:1, we still have no evidence for the characteristic of sex-controlled inheritance. The onset age of the disease is very early with a median age of 6 months, and the morbidity rate was 75% before 1 year of age. As an autosomal recessive disease, the parents of HTGTI patients showed no related symptoms, but consanguineous marriage may be a high-risk factor for the disease. Therefore, we should be alert to the possibility of HTGTI when some infants and children are accompanied by unexplained HTG, hepatosplenomegaly, and abnormal liver function.

It is generally considered that HTG caused by the *GPD1* variant is temporary, which will gradually become regular with age. However, the analysis of the laboratory data of TG provided by these patients showed that only 30% of the patients recovered to normal from HTG during follow-up. The remaining patients still had different degrees of HTG during the follow-up period. This abnormal situation even lasted until the age of 31 years. Our fitting curve also shows that HTG in early infants decreases rapidly with age, especially within 1 year of age. However, its average level still exceeded the normal upper limit during the later period. Meanwhile, some children were still accompanied by continuous mild to moderate fatty liver, elevated transaminase, and liver cirrhosis. Therefore, the characteristic of mild or moderate persisting HTG in some patients indicates that it is a prolonged process rather than temporary. This also reflects the persistent disorder of lipid metabolism, which may have a long-term adverse effect on health. Epidemiological survey data show that the morbidity of HTG in adults was 10–20% (Parhofer and Laufs 2019; Laufs et al., 2020), but there are no relevant investigation data of infants for use. At the same time, the overall prevalence of NAFLD in children was approximately 3–10% (Anderson et al., 2015). In particular, many studies indicate that HTG is an independent or increased risk factor for atherosclerosis, coronary heart disease, diabetes, acute pancreatitis, and even tumors (Cullen 2000; Huang et al., 2016; Packard et al., 2020). These findings may help us better understand the disease and maintain more sustained attention to dyslipidemia in childhood.

Dyslipidemia is the most common lipid metabolism disorder. However, its exact etiology and pathogenic mechanisms remain elusive. The occurrence of HTG results from either increased synthesis in the liver or decreased TG degradation and uptake. The HTG phenotype is regulated by the complex networks of multiple gene variation and secondary factors (Hegele et al., 2014; Lewis et al., 2015; Liu et al., 2019). Previous studies have demonstrated that monogenic HTG in patients with severe HTG (TG > 10 mmol/L) displays classic autosomal recessive hereditary disorders. Affected individuals are often homozygous or compound heterozygous for large-effect loss-of-function variants in genes that regulate catabolism of TG-rich lipoproteins, such as *LPL* (Henderson et al., 1991), *APOC2* (Baggio et al., 1986), *APOA5* (Kao et al., 2003), *LMF1* (Peterfy et al., 2007), *GPIHBP1* (Wang and Hegele 2007), and *GPD1* (Hegele et al., 2014; Liu et al., 2019).

The human *GPD1* gene is located on chromosomal region 12q12-q13 (RefSeq: NM_005276.2) and encodes cytoplasmic NAD-dependent GPD1. The open reading frame of mRNA, including eight coding exons, encodes a protein of 349 amino acid residues, which catalyzes the reversible biological conversion of dihydroxyacetone phosphate (DHAP) to glycerol-3-phosphate (G3P). The variants in the *GPD1* gene have been reported to cause HTGTI in these 31 patients. The primary variant sites of the *GPD1* gene were missense variants (51.6%), followed by splicing variants (35.5%) and nonsense variants (12.9%). Research has shown that the splice-site homozygous variant in intro 3 (c.361-1G > C) creates a truncated protein of 213 residues and missing some major secondary structures and active sites (Basel-Vanagaite et al., 2012). The compound heterozygous missense variant in exon 6 (c.686G > A) is associated with a lack of GPD1 protein and a reduction in CPT1 and CPT2 activity (Joshi et al., 2014). Another compound heterozygous missense variant in exon 6 (c.820G > A) and splicing variant in exon 3 (c.220–2A > G) generated a decreased expression of the protein and the loss of bases (Li et al., 2017). The crystal structure of GPD1 in Rao’s study showed that Arg229 provides 3 of 11 hydrogen bonds in the dimer interface. Arg269, as a conserved active site of the ternary complex of GPD1/DHAP/NAD^+^, is in direct contact with the substrate, while Gly299 constitutes the other fixed point of the three-dimensional structure of the binary complex of GPD1 with NAD^+^(Ou et al., 2006). Therefore, variants at these loci resulted in the loss or low activity of the GPD1 enzyme, which further led to the disorder of glucose and lipid metabolism.

The deep investigation of monogenic dyslipidemias has the potential to reveal previously unrecognized key pathways in lipid metabolism. HTGTI is caused by the inactivation and variant of *GPD1*, which plays a critical role in carbohydrate and lipid metabolism. The *GPD1* gene variant is one of the significant molecular etiologies of primary HTG with the onset of disease in infancy (Basel-Vanagaite et al., 2012). Basel-Vanagaite et al. (2012) confirmed that the transience of HTG might follow the pattern of hepatocyte triglyceridemia, that is, a higher secretion rate in the newborn than in adults. However, the exact mechanism behind the multiple clinical characteristics and pathogenesis of the disease remains largely unknown, which needs to be further clarified. At present, there is no satisfactory therapeutic approach available for this inherited disorder. The Summary Report of the Expert Panel on Integrated Guidelines for Cardiovascular Health and Risk Reduction in Children and Adolescents advises that people aged 10–21 years with lipid abnormalities (LDL cholesterol level of ≥250 mg/dl and/or TG level of >500 mg/dl) should be handled for 3–6 months with diet adjustments (Expert Panel on Integrated Guidelines for Cardiovascular Health and Risk Reduction in Children and Adolescents; National Heart, Lung, and Blood Institute, 2011). A diet rich in low-fat medium-chain TG or antihyperlipidemic drugs may be effective in some people for lowering TG levels. Sometimes, pharmacological therapy should be considered for children and adolescents (Fiorentino and Chiarelli 2021), while efficacy and safety data for triglyceride-lowering drugs are still prudent under 18 years old (Valaiyapathi et al., 2017; Fiorentino and Chiarelli 2021).

Owing to the absence of specific biochemical markers, the underdiagnosis of HTGTI is quite possible in children or adolescents with hepatomegaly or hepatopathy of unknown origin or nonalcoholic steatohepatitis (NASH) (Tesarova et al., 2021). In recent years, with the application of gene sequencing in the diagnosis of rare diseases, more and more hereditary metabolic diseases have been confirmed and gained effective treatment and prognostic guidance. Meanwhile, it has also increased the awareness of the features and mechanisms of these sorts of diseases, for example, the diagnosis and treatment of HTGTI have also benefited a lot from it, and new cases are being discovered constantly (Lin et al., 2021). We believe that our understanding about HTGTI will gradually grow in the future. However, it is necessary to update the knowledge that dyslipidemia may not be transient in infancy, and HTG may rebound and present long-lasting abnormality with mild to moderate levels for the majority of these patients. Furthermore, cirrhosis developing at such a young age in some children opposes the prevailing perception of GPD1 being a benign or transient disease. Therefore, some specialists advise that the original name of the disease, “transient infantile hypertriglyceridemia,” should be abandoned because it cannot precisely reflect the condition of the patient (Tesarova et al., 2021).

## Conclusion

Although severe HTG is transient at the early age of infants for some patients, it may not maintain normal with age spontaneously for most of these patients. Meanwhile, persistent mild HTG and liver damage indicate long-lasting detrimental effects on the health of patients rather than transience. However, as the specific signs and symptoms for diagnosis and the long-term follow-up data are still unavailable, the clinical manifestations and molecular pathogenesis of HTGTI are not entirely understood. Therefore, a lipid-clinic evaluation and a close follow-up are recommended for the patients throughout their life.

## Data Availability

The original contributions presented in the study are included in the article/Supplementary Material, and further inquiries can be directed to the corresponding author.
